# Evaluation of the cross-sectional area of small pulmonary vessels in the diagnosis of chronic obstructive pulmonary disease by quantitative computed tomography: A case-control study

**DOI:** 10.1097/MD.0000000000027622

**Published:** 2021-11-19

**Authors:** Yifan Wang, Tong Su, Shaotong Feng, Juan Chen, Xingcang Tian, Li Zhu

**Affiliations:** a Department of Radiology, General Hospital of Ningxia Medical University, Ningxia, China; b Department of Pneumology, General Hospital of Ningxia Medical University, Ningxia, China.

**Keywords:** chronic obstructive pulmonary disease, computed-tomography, Image J, quantitative assessment, small pulmonary vessels

## Abstract

Patients with chronic obstructive pulmonary disease (COPD) have a reduced cross-sectional area (CSA) of small pulmonary vessels and decreased pulmonary function test (PFT) indexes. This study investigated the value of small pulmonary vessel CSA in diagnosing and evaluating the severity of COPD and its correlation with PFT.

This retrospective case-control study included patients with COPD who underwent multi-slice spiral computed tomography (CT) between March 2015 and December 2018. COPD severity was graded. Patients with normal CT results were included as controls. The CSA of small pulmonary vessels at the sub-segmental (5–10 mm^2^) and sub-sub-segmental (<5 mm^2^) levels was measured. Receiver operating characteristic (ROC) curves were used to evaluate the effect of CSA for COPD risk prediction. The correlation between CSA% and PFT indexes was evaluated.

There were 124 and 106 patients in the COPD and control groups, respectively. The %CSA <5 and %CSA5–10 were smaller in the COPD group than in controls (*P* *<* .05). The %CSA <5 in each subgroup stratified by COPD severity was smaller than in controls (*P* *<* .05). The % CSA5–10 was significantly smaller in the moderate and severe groups than in controls (*P* *<* .05). At 0.655%CSA <5 cut-off, the ROC area under the curve (AUC) was 0.765. For %CSA5–10, a 0.565 cut-off led to an AUC of 0.752. Both %CSA <5 and %CSA5–10 were positively correlated with all PFT indexes (*r* = 0.180–0.462, all *P* *<* .05).

CSA was positively correlated with PFT. Analysis of small pulmonary vessel CSA based on CT images contributes to diagnosing and assessing the severity of COPD.

## 1 Introduction

The Global Initiative for Chronic Obstructive Lung Disease (GOLD) defines chronic obstructive pulmonary disease (COPD) as a common, preventable, and treatable disease.^[1]^ COPD is a complex disease characterized by limited airflow, inflammation, and changes in blood vessels.^[2]^ COPD manifests as obstructive bronchiolitis, emphysema, chronic mucus secretion, and small airway thickening,^[3]^ which seriously affect the patient's quality of life. The morbidity and mortality of COPD have been increasing each year through the 21st century.^[4]^ The early diagnosis and evaluation of the severity of the disease are vital in the selection of treatment options and prognosis of COPD patients.^[5]^

At present, the main examination method of COPD is a pulmonary function test (PFT)^[6]^ that involves measuring the forced expiratory volume in the first second (FEV1), forced vital capacity (FVC), and FEV1/FVC. Still, some studies concluded that FEV1 does not fully reflect the full clinical status of patients with COPD.^[7]^ Besides, PFT does not fully reflect the severity of shortness of breath, limited activity, and health damage in patients.^[1]^

Alongside airway stenosis,^[8]^ small pulmonary vascular change is one of the main pathological changes and important risk factors for COPD progression.^[9]^ For some COPD patients without severe airflow obstruction or emphysema, there can also be a significant reduction in the cross-sectional area (CSA) of small pulmonary vessels.^[2]^ A recent study using gadolinium-enhanced magnetic resonance imaging showed that the pulmonary microvascular blood flow might also be reduced when there is no clear emphysema, suggesting that developing emphysema might correlate with pulmonary vascular remodeling.^[9]^ Therefore, the measurement of the CSA of small blood vessels by quantitative computed tomography (CT) has been introduced to reflect the changes in small pulmonary vessels during COPD progression.^[10,11]^ It is the preferred method to evaluate COPD pulmonary arteriolar lesions. Without special CT scanning technology and contrast agent injection, it is a relatively simple method and the ideal noninvasive imaging method for studying small pulmonary vessels.^[11]^ Nevertheless, in patients with COPD, there have been many studies on airway changes and emphysema by PFT, but studies on vascular changes, especially of the small pulmonary vessel by the measurement of CSA, are rare.

Therefore, this study aimed to use multi-slice spiral CT images to explore the use of small pulmonary vessel CSA in diagnosing and evaluating the severity of COPD and its correlation with PFT.

## 2 Methods

This retrospective study was approved by the ethics committee for human research of the General Hospital of Ningxia Medical University [2020–03]. The requirement for individual informed consent was waived by the committee.

### 2.1 Subjects

Patients who underwent multi-slice spiral CT examination at the Department of Radiology of the General Hospital of Ningxia Medical University from March 2015 to December 2018 and who were clinically diagnosed with COPD according to the 2015 edition of the GOLD strategy^[12]^ were retrospectively collected as the COPD group. The patients in the control group were from the same hospital, department, and study period as the COPD group.

The inclusion criteria for the COPD group were

1. COPD was confirmed by clinical history and PFT,^[12]^ and
2. patients with complete clinical information.

Patients were excluded for

1. CT images with serious image artifacts or poor-quality images,
2. other underlying lung diseases (including thoracic deformity, many pleural effusions, widespread infection or consolidation, severe pulmonary fibrosis, and lung cancer), or
3. had undergone chest surgery, such as lung volume reduction surgery.

The patients were included in the control group if they underwent CT examination and were without COPD and were excluded if they had a history of

1. heart failure,
2. coronary heart disease, or
3. previous pneumonectomy.

### 2.2 Pulmonary function tests

The patients were examined by PFT and MSCT successively, and the interval between the 2 examinations did not exceed 7 days. In this study, the American Thoracic Association criteria were used as the standard for measuring respiratory volume.^[13]^ FEV1, FVC, the percentage of the measured value in the estimated value (FEV1%), and the ratio of FEV1 to FVC% were obtained. The severity of COPD was divided into 3 subgroups according to the PFT results.^[12]^ The mild patients were classified as GOLD I: FEV1/FVC <70% and FEV1 ≥80% predicted. The moderate group was classified as GOLD II: FEV1/FVC <70% and 50%≤ FEV1 <80% predicted. The severe group was classified as GOLD III/IV: FEV1/FVC <70% and FEV1 <50% predicted.

### 2.3 Multi-slice computed tomography scanning

All patients underwent a chest plain scan with a 16-detector scanner (Brilliance 16. Philips, Netherlands) in the supine position. The patients underwent breathing training before scanning, and the whole lung was scanned from the apex to the bottom of the lung at the end of quiet inspiration. The scanning parameters were tube voltage 120 kV, tube current 106 mAs, collimator 16 × 0.5 mm, frame rotation time 0.75 s/r, pitch 1.0, and matrix 512 × 512. The reconstruction thickness was 2 mm, and the interval was 1 mm. Two radiologists with 10 years of experience in thoracic radiology (YW and LZ) reviewed all chest CT scans.

### 2.4 Computed tomography measurement of small pulmonary vessels

For the measurements of small pulmonary vessel CSA, 3 CT slices were selected. The upper cranial slice was taken 1 cm above the upper margin of the aortic arch, the middle slice was taken 1 cm below the carina, and the lower caudal slice was taken 1 cm below the right inferior pulmonary vein. The CT images were analyzed using a semiautomatic image-processing program (Image J 1.37 bundled with Java image processing program available on the Web at http://rsb.info.nih.gov/ij/). Using the “Analyze Particles” function of the Image J software that can count and measure objects on binary images, the number and CSA of each vessel on each CT slice were obtained.

**Figure 1:**
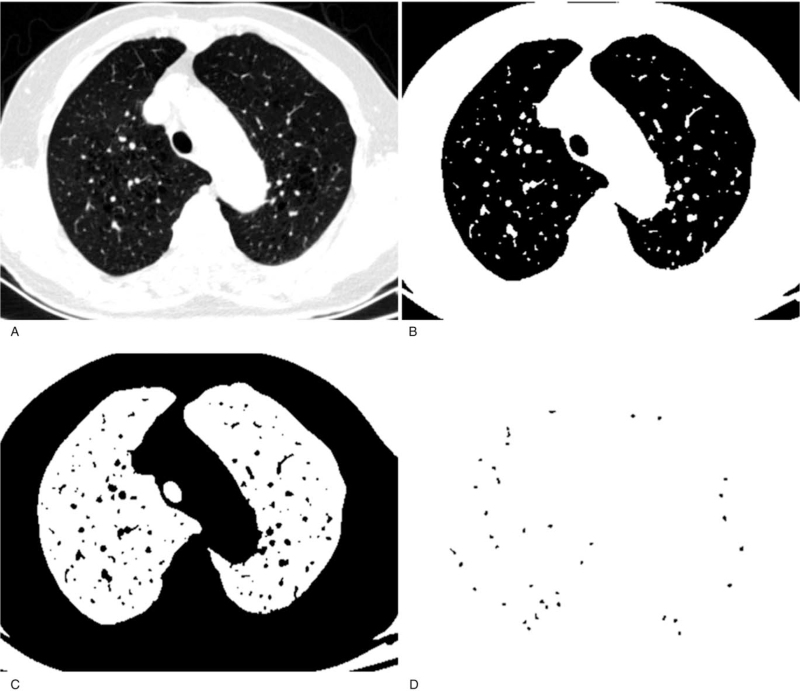
Measurement of the CSA of small pulmonary vessels. (A): The CT image shows the lung field segmented within threshold values from −500 to −1024 HU. (B): CT image segments in (A) with a window level of −720 HU. (C): Binary image converted from (B). (D): Mask image was obtained for a particle analysis after setting the vessel size parameters to within 0 to 5 mm^2^. CSA = cross-sectional area.

CSA measurements were conducted as follows (Fig. 1). First, the lung field was segmented using the threshold technique with all pixels between −500 and −1024 HU on each CT image. Next, the segmented images were converted into binary images with a window level of −720 HU, and the vessels were then displayed in black on the binary image. We separately measured the CSA at the sub-segmental and sub-sub-segmental levels: the range of CSA of each vessel was defined at less than 5 mm^2^ at the sub-sub-segmental level and 5 to 10 mm^2^ at the sub-segmental level. Using these settings, the CSA for each vessel range was calculated. Finally, the total CSA of the vessels was calculated on each set of 3 CT slices, and those totals were abbreviated as follows: CSA <5 for the number of vessels that were less than 5 mm^2^ and CSA5–10 for the number of vessels that were between 5 and 10 mm^2^. The total area of the lung in the selected 3 slices was obtained using threshold values between −500 HU and −1024 HU, and the percentages of CSA <5 (%CSA <5) and CSA5–10 (%CSA5–10) for the total area of the lung were calculated.

### 2.5 Statistical analysis

The area of small pulmonary vessels was analyzed using Image J software (https://imagej.nih.gov). The statistical data analysis was undertaken using SPSS 21.0 (IBM Corp, Armonk, NY). The categorical variables were analyzed using the Chi-Squared test. Measurement data were expressed as mean ± standard deviation (SD). An independent sample *t*-test was used to analyze the differences between the COPD and control groups. Univariable analysis of variance (ANOVA) was used to compare the light, moderate, and severe COPD groups with the control group, and the Scheffe test was used for pairwise comparison of the differences between the groups with statistical significance. The area under the receiver operating characteristic (ROC) curve (AUC) was used to evaluate the effect of CSA% for COPD prediction. Spearman correlation was used to analyze the relationship between patient %CSA and PFT. *P* *<* .05 was considered statistically significant.

## 3 Results

### 3.1 Patient characteristics

The COPD group included 124 patients aged 65.8 ± 9.3 years; there were 80 (64.5%) males and 44 (35.5%) females. The control group included 106 patients aged 60.7 ± 8.1 years; there were 48 (45.3%) males and 58 (54.7%) females. The general data of the COPD and control groups are shown in Table 1. There were differences between the groups in age (*P* = .003) and gender (*P* *<* .001), but body mass index was similar (*P* > .05).

**Table 1:** Patient characteristics in COPD group and control group.

Characteristics	COPD group (n = 124)	Control group (n = 106)	*P*
Sex ratio (Male/Female)	64.5%/35.5%	45.3%/54.7%	.003
Age (yr)	65.8 ± 9.3	60.7 ± 8.1	<.001
BMI	25.3 ± 14.7	24.1 ± 3.4	.379

COPD = chronic obstructive pulmonary disease, BMI = body mass index. *P* *<* .05 was considered statistically significant.

### 3.2 Comparison of % cross-sectional area between groups

**Figure 2:**
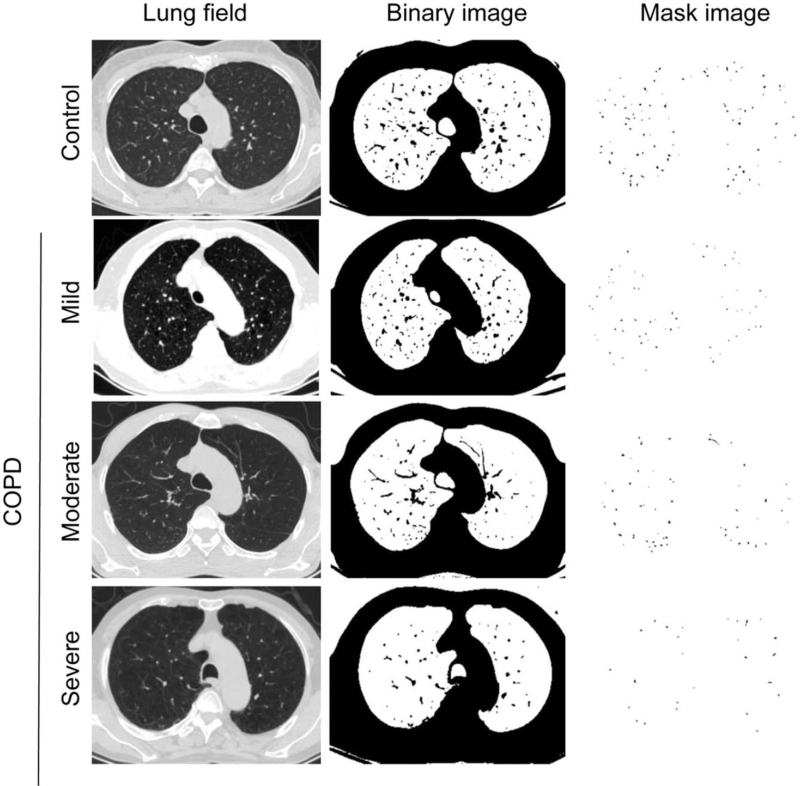
Example CT images for %CSA <5 in different COPD groups. The CT image of %CSA <5 in the control, mild, moderate, and severe groups (from top to bottom), are shown. %CSA <5: the ratio of the sum of the sectional area of pulmonary small blood vessels with the sectional area <5 mm^2^ to the pulmonary sectional area; COPD = chronic obstructive pulmonary disease.

As shown in Figure 2, the pulmonary small blood vessels decreased with the increasing severity of COPD. The %CSA <5 and %CSA5–10 values in the COPD group were smaller than in the control group (*P* *<* .05). When the patients were classified into subgroups according to the severity of COPD, the %CSA <5 values were smaller in every subgroup compared to the control group (*P* *<* .05). The %CSA5–10 values were not significantly different between the mild and control groups (*P* > .05), but they were significantly smaller in the moderate and severe groups compared with the control group (*P* *<* .05) (Table 2).

**Table 2:** Comparison of %CSA of small pulmonary vessels between groups graded according to severity of COPD.

		COPD group (n = 124)			
%CSA	Control group (n = 106)	Mild group (n = 35)	Moderate group (n = 45)	Severe group (n = 44)	*P*
<5, %	0.87 ± 0.24^∗^^,^^&^	0.76 ± 0.24^#^	0.67 ± 0.19^#^^,^^∗^	0.49 ± 0.11^#^^,^^∗^^,^^&^	<.001
5–10, %	0.65 ± 0.17^&^	0.64 ± 0.17	0.52 ± 0.15^#^	0.41 ± 0.09^#^^,^^∗^^,^^&^	<.001

%CSA = ratio of pulmonary small blood vessels to lung sectional area, %CSA<5 = the ratio of the sum of the sectional area of pulmonary small blood vessels with the sectional area <5 mm^2^ to the pulmonary sectional area, %CSA5–10 = ratio of the sum of the sectional areas of pulmonary small blood vessels with a sectional area between 5 and 10 mm^2^ to the pulmonary sectional area, COPD = chronic obstructive pulmonary disease.

# Represents *P* *<* .05 compared with the control group.

∗ Represents *P* *<* .05 compared with the mild group, and.

& Represents *P* *<* .05 compared with the moderate group. *P* *<* .05 was considered statistically significant.

The %CSA<5 and %CSA5–10 values continued to decrease gradually with severity through the 3 COPD subgroups, as shown in Table 2. The pairwise comparisons of the differences in %CSA among groups with different severity of COPD showed significant differences in %CSA<5 among the mild, moderate, and severe groups (*P* *<* .05). For %CSA5–10 values, there was a significant difference between the mild and severe groups and between the moderate and severe groups (*P* *<* .05), but not between the mild and moderate groups (*P* > .05).

### 3.3 The value % cross-sectional area to diagnose chronic obstructive pulmonary disease

**Figure 3:**
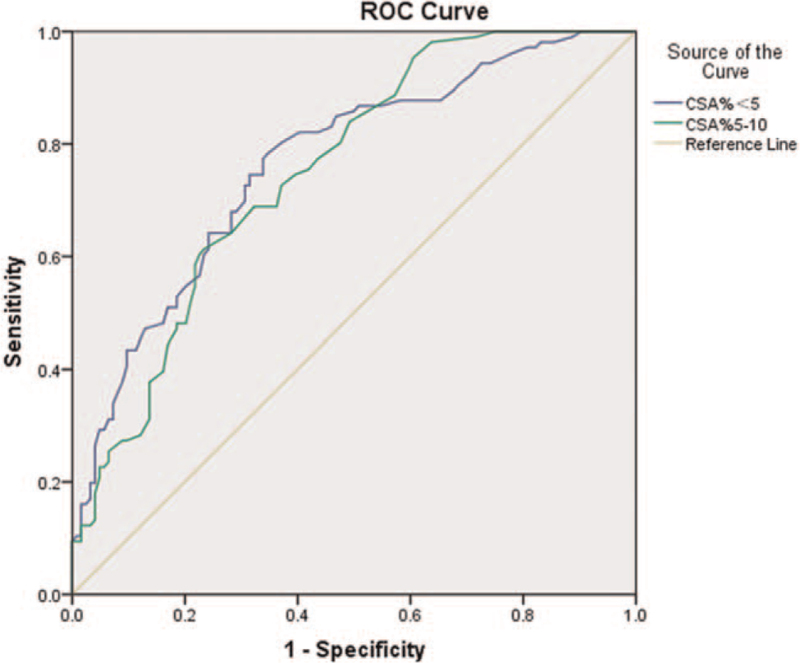
ROC curve analysis of CSA% to predict COPD. At 0.655%CSA <5 cut-off, the AUC was 0.765, sensitivity was 78.3%, specificity 65.3%, and the Youden index was 0.436. For %CSA5–10, at a 0.565 cut-off, the AUC was 0.752, sensitivity was 61.3%, specificity was 76.6%, and the Youden index was 0.379. ROC = receiver operating characteristic, AUC = area under the curve, %CSA = ratio of pulmonary small blood vessels to lung sectional area, %CSA<5 = the ratio of the sum of the sectional area of pulmonary small blood vessels with the sectional area <5 mm^2^ to the pulmonary sectional area, %CSA5–10 = ratio of the sum of the sectional areas of pulmonary small blood vessels with a sectional area between 5 and 10 mm^2^ to the pulmonary sectional area, COPD = chronic obstructive pulmonary disease.

The ROC curves of %CSA <5 and %CSA5–10 were drawn to determine the ability of these parameters to diagnose COPD (Fig. 3). All curves were above the reference line, and the AUC of %CSA <5 was slightly larger. At a cut-off of 0.655 for %CSA <5, the AUC was 0.765, sensitivity was 78.3%, specificity was 65.3%, and the Youden index was 0.436. For %CSA5–10, at a cut-off of 0.565, the AUC was 0.752, sensitivity was 61.3%, specificity was 76.6%, and the Youden index was 0.379.

### 3.4 Correlation between pulmonary function test and % cross-sectional area in the chronic obstructive pulmonary disease subgroups

The comparisons of the pulmonary function indexes in the control group and COPD severity subgroups are shown in Table 3. The FVC, FEV1, FEV1%, and FEV1/FVC% of the COPD group were lower than in the control group (*P* *<* .001). Pairwise comparisons showed that FEV1, FEV1%, and FEV1/FVC% were significantly higher in the mild and control groups (all *P* *<* .05), but there was no statistically significant difference in FVC (all *P* > .05). All PFT indexes decreased with COPD severity and were the lowest in the severe group, with significant differences in all indicators among the moderate, severe, and control groups (all *P* *<* .05), and significant differences in all indicators among the mild, moderate, and severe groups (all *P* *<* .05).

**Table 3:** Comparison of PFT between groups.

		COPD group (n = 124)			
Indexes	Control group (n = 106)	Mild group (n = 35)	Moderate group (n = 45)	Severe group (n = 44)	*P*
FVC(L)	3.49 ± 0.74	3.39 ± 1.01^&^	2.85 ± 0.92^#^^,^^∗^	2.49 ± 0.65^#^^,^^∗^^,^^&^	<.001
FEV1(L)	2.79 ± 0.59	2.17 ± 0.73^#^^,^^&^	1.56 ± 0.53^#^^,^^∗^	0.94 ± 0.28^#^^,^^∗^^,^^&^	<.001
FEV1%	111.66 ± 14.83	89.75 ± 9.62^#^^,^^&^	63.58 ± 11.72^#^^,^^∗^	38.07 ± 8.08^#^^,^^∗^^,^^&^	<.001
FEV1/FVC%	80.18 ± 3.76	62.16 ± 8.24^#^^,^^&^	56.18 ± 9.48^#^^,^^∗^	37.16 ± 8.09^#^^,^^∗^^,^^&^	<.001

COPD = chronic obstructive pulmonary disease, FVC = forced vital capacity, PFT = pulmonary functiontest, FEV1 = forced vital volume in the first second, FEV 1% = the percentage of measured value in the estimated value, FEV1/FVC% = the ratio of FEV1 to FVC%.

# Represents *P* *<* .05 compared with the control group.

∗ Represents *P* *<* .05 compared with the mild group, and.

& Represents 0.05 compared with the moderate group. 0.05 was considered statistically significant.

**Figure 4:**
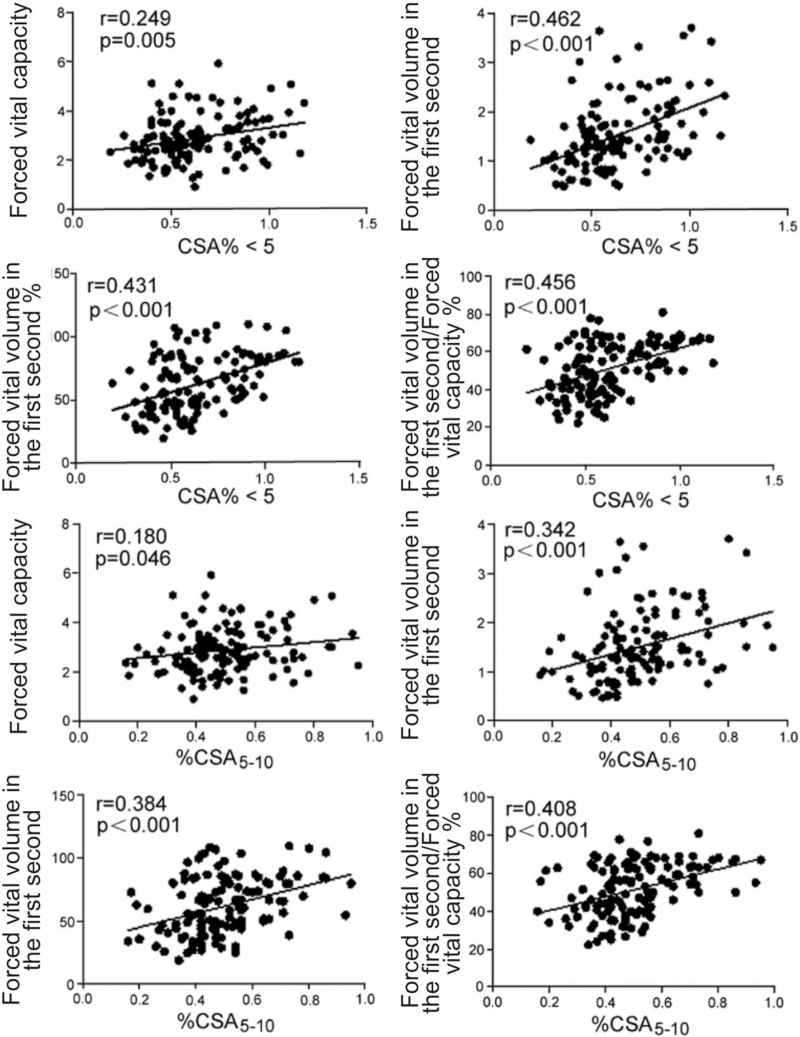
Correlations between %CSA and PFT in different COPD groups. The %CSA = ratio of pulmonary small blood vessels to lung sectional area, %CSA<5 = the ratio of the sum of the sectional area of pulmonary small blood vessels with the sectional area <5 mm^2^ to the pulmonary sectional area, %CSA5–10 = ratio of the sum of the sectional areas of pulmonary small blood vessels with a sectional area between 5 and 10 mm^2^ to the pulmonary sectional area, COPD = chronic obstructive pulmonary disease, PFT = pulmonary function test, FVC = forced vital capacity, FEV1 = forced vital volume in the first second, FEV 1% = the percentage of measured value in the estimated value, FEV1/FVC% = the ratio of FEV1 to FVC%. *P* *<* .05 was considered statistically significant.

As shown in Figure 4, %CSA <5 was significantly positively correlated with FVC (*r* = 0.249, *P* = .005), FEV1 (*r* = 0.462, *P <* .001), FEV1% (*r* = 0.180, *P* *<* .001), and FEV1/FVC% (*r* = 0.456, *P* *<* .001). %CSA5–10 was also significantly positively correlated with FVC (*r* = 0.180, *P* = .046), FEV1 (*r* = 0.342, *P* *<* .001), FEV1% (*r* = 0.384, *P* *<* .001), and FEV1/FVC% (*r* = 0.408, *P* *<* .001). The analysis suggested that the correlation between %CSA <5 and PFT was stronger than for %CSA5–10, and the difference between %CSA <5 and FEV1 had the greatest significance.

## 4 Discussion

The results showed significant differences in %CSA <5 and %CSA5–10 between the COPD and control groups and decreases in these values with increasing COPD severity. The ROC analysis suggested that a cut-off of 0.655 for %CAS <5 had an AUC of 0.765, while %CAS5–10 at a cut-off of 0.565 had an AUC of 0.752. There were significant correlations between small pulmonary vessel CSA, including %CSA <5 and %CSA5–10, and the 4 PFT indexes. Therefore, these results suggest that CSA can be useful in assisting the diagnosis of the severity of COPD.

Previous studies showed that it is possible to measure %CSA based on CT images to reflect changes in pulmonary microvessels.^[10,11,13]^ In this study, the CSA was separately measured at 2 levels: %CSA <5 and %CSA5–10. The results showed that the %CSA <5 and %CSA5–10 values of COPD patients were lower than in the control group. In addition, both measures of %CSA decreased with increasing COPD severity, suggesting increasing vascular damage with increasing COPD severity. It suggests that CSA might be an indicator used to evaluate the severity of COPD, especially %CSA<5, which is supported by previous studies.^[14–16]^ Nevertheless, a previous study of %CSA5–10 and COPD found that while %CSA5–10 values were decreased in COPD, there were no associations with severity.^[14]^ It could be due to differences in study populations and sample size. Thus, %CSA <5 might have a higher value in COPD severity prediction than %CSA5–10. %CSA<5 might also be more useful as a diagnostic tool for COPD. Furthermore, the AUC value of %CSA5–10 to diagnose COPD was 0.752, but a higher value was achieved with %CSA <5, which was 0.765, with a diagnostic sensitivity of 78.3%, but the specificity was 65.3%. Therefore, it is feasible to predict COPD with the %CSA <5 indexes, but the specificity is low, suggesting that screening for COPD with %CSA <5 might result in a high number of false-positive tests. It supports the use of %CSA <5 to predict acute exacerbation of COPD that achieved AUC of 0.764 in a previous study.^[14]^ Since PFT alone does not fully reflect the severity of shortness of breath, limited activity, and health damage in patients,^[1]^ CSA could be used as an auxiliary test for the diagnosis and grading of COPD. All patients with COPD usually undergo CT as a routine examination, and the determination of CSA requires software that is available on the Internet. Hence, it could be used to improve COPD diagnosis, especially in patients without severe airflow obstruction or emphysema, since such patients display a significant reduction in the CSA of pulmonary small vessels.^[2]^

Airflow obstruction in COPD patients is affected by both small airway disease and emphysema, and these are indicated by different measurements in PFT. For example, the decrease in FEV1 is closely related to functional small airway disease and declines at its highest rate early in mild to moderate COPD.^[17]^ The present study showed that FVC, FEV1, FEV1%, and FEV1/FVC% were all decreased in patients with COPD, and the values were lower as the severity of COPD increased, except that there was no significant difference between the mild and control groups for FVC. In addition, there were positive correlations between both %CSA <5 and %CSA5–10 with the 4 PFT indexes. The correlation between %CSA <5 and PFT was stronger than that of %CSA5–10, with the largest significant difference being between %CSA <5 and FEV1. These results further demonstrated that the CSA of small pulmonary vessels was closely related to pulmonary function indicators in COPD patients, especially %CSA <5, which is supported by a previous study that found a significant but weaker correlation of %CSA <5 with FEV1.^[15]^

One of the important factors for the quantitative analysis of small pulmonary vessels is selecting the size of small pulmonary vessels. The %CSA <5 can be considered to reflect elastic blood vessels and muscular blood vessels, while %CSA5–10 are mainly elastic blood vessels. The vascular changes in patients with COPD mainly occur in muscular pulmonary arteries. Hypoxic alveoli redistribute blood to the best-ventilated lung segments, especially small muscular pulmonary arteries, through vasoconstriction.^[18]^ Therefore, small pulmonary vessel changes in patients with COPD mainly involve small blood vessels below the sub-segment.^[16]^ Matsuoka et al^[10]^ reported that %CSA <5 was significantly correlated with the severity of emphysema, but not %CSA5–10. Wang et al^[14]^ reported that %CSA <5 was significantly reduced in patients with acute COPD and confirmed that %CSA <5 was a good index for evaluating acute COPD. Our results support this concept because although %CSA5–10 was correlated with lung function, %CSA <5 was more effective in reflecting COPD severity.

This study has some limitations. As a retrospective case-control study, the sample size was small. In addition, this study did not separate smokers and nonsmokers, and it is known that smoking can directly damage blood vessels and cause pulmonary vascular remodeling.^[19]^ In addition, the sex distribution and age of the COPD and control groups were different, mainly because age and sex are risk factors for COPD, so this might have introduced some bias. Some authors believe that the aging of airway and lung parenchyma is similar to some structural changes related to COPD.^[20]^ Hospitalized patients tended to be seriously ill, and the number of mild and severe COPD cases was small, possibly leading to small differences in some parameters between the mild and control groups. In future research, a larger sample size will be necessary.

In conclusion, this study revealed correlations between CSA and PFT, as well as the potential value of pulmonary microvascular CSA in the diagnosis of COPD and in reflecting the severity of COPD by quantitative CT. Further studies are needed to provide more valuable imaging information for the diagnosis and classification of COPD.

## Author contributions

**Data curation:** Tong Su, Xingcang Tian.

**Formal analysis:** Shaotong Feng, Juan Chen.

**Funding acquisition:** Li Zhu.

**Methodology:** Shaotong Feng, Juan Chen.

**Software:** Xingcang Tian.

**Supervision:** Tong Su.

**Writing – original draft:** Yifan Wang.

**Writing – review & editing:** Li Zhu.
